# Treatment with Remdesivir of Children with SARS-CoV-2 Infection: Experience from a Clinical Hospital in Romania

**DOI:** 10.3390/life14030410

**Published:** 2024-03-20

**Authors:** Maria-Elena Cocuz, Iuliu Gabriel Cocuz, Ligia Rodina, Elena Tataranu, Olga Adriana Caliman-Sturdza, Florin Filip

**Affiliations:** 1 Fundamental Prophylactic and Clinical Disciplines Department, Faculty of Medicine, Transilvania University of Brasov, 500003 Brasov, Romania; maria.cocuz@unitbv.ro; 2 Clinical Pneumology and Infectious Diseases Hospital of Brasov, 500118 Brasov, Romania; ligiarodina@yahoo.com; 3 Pathophysiology Department, “George Emil Palade” University of Medicine, Pharmacy, Sciences and Technology of Targu Mures, 540142 Targu Mures, Romania; 4 Pathology Department, Mures Clinical County Hospital, 540011 Targu Mures, Romania; 5 Clinical Department of Pediatrics, “Sf. Ioan cel Nou” Emergency Hospital, 720224 Suceava, Romania; elena.tataranu@usm.ro; 6 Department of Infectious Diseases, “Sf. Ioan cel Nou” Emergency Hospital, 720224 Suceava, Romania; olga.caliman-sturdza@usm.ro; 7 “Sf. Ioan cel Nou” Emergency Hospital, 720224 Suceava, Romania; florin.filip@usm.ro; 8 Faculty of Medicine and Biological Sciences, Stefan cel Mare University of Suceava, 720229 Suceava, Romania

**Keywords:** remdesivir, COVID-19, children, SARS-CoV-2

## Abstract

Background: The COVID-19 pandemic was characterized by mild-to-moderate disease in children and adolescents, with low incidences of severe cases and mortality. Most of the information on drug therapy in COVID-19-positive children was derived from research in adult patients. Remdesivir, an inhibitor of viral RNA polymerase, was shown to be effective in COVID-19 patients with moderate-to-severe disease. In this study, we present our experience of the use of remdesivir in pediatric patients hospitalized with COVID-19. Materials and methods: This retrospective study was based on the early use of remdesivir in 14 children with mild, moderate, and severe clinical forms of COVID-19, who were hospitalized between 1 January 2022, and 30 September 2023. Results: The patients included eight infants and six children older than 1 day (the age range was 2 months to 17 years). Most of them (92.85%) had documented pneumonia. Four patients had associated acute laryngitis, and another had bronchiolitis. Coinfections with Streptococcus pneumoniae were diagnosed in two patients. The clinical course was favorable in 12/14 (85.71%) children. Two patients were transferred to the pediatric intensive care unit because of aggravation of associated acute diseases (acute laryngitis and bronchiolitis, respectively). Mild increases in alanine aminotransferase levels occurred in two patients, with no increase in serum creatinine, during treatment with remdesivir. Conclusion: The appropriate use of remdesivir proved safe and efficient in our group of patients. However, further studies are required to support the efficiency, tolerability, and safety of remdesivir in children.

## 1. Introduction

The coronavirus 2019 (COVID-19) pandemic represented a serious challenge to healthcare systems worldwide. Children and adolescents experienced less severe disease compared to adults, with most cases requiring only symptomatic treatment or no treatment [1,2]. Mortality at young ages was related to certain risk factors and had a low incidence [3,4,5]. The choice of the appropriate therapy for children was a significant issue during the pandemic. Few clinical studies have examined drug therapy for children and adolescents with SARS-CoV-2 infection; the limited number of studies available have generally included adult patients [6,7,8,9,10].

Remdesivir, a nucleotide prodrug analog that inhibits viral RNA polymerase, displays in vitro and in vivo activity against SARS-CoV-2 [1,11,12]. It has shown clear benefits in moderate-to-severe pediatric cases of COVID-19, especially for children of all ages with an increase in supplemental oxygen [13]. The use of remdesivir has been approved for pediatric patients by the US Food and Drug Administration and for adolescent patients (≥12 years) by the European Medicines Agency (EMA) [14,15]. The criteria for applying remdesivir treatment are a weight of at least 40 kg and age of at least 12 years, the onset of disease a maximum of 7 days ago, the lack of need for additional oxygen, and the presence of risk factors for severe disease evolution [16]. In most cases, treatment with remdesivir has been evaluated in randomized clinical trials with adults. Regarding the safety of using remdesivir, a study carried out with adults highlighted biological changes, represented by increased serum creatinine and serum alanine aminotransferase values, as well as clinical side effects, namely, bradycardia, in addition to the mortality benefit [17,18]. Experience with the safety and efficacy of remdesivir use in pediatric patients is limited [19]. This article presents our experience with the use of remdesivir in 14 pediatric patients admitted with SARS-CoV-2 infection between 1 January 2022, and 30 September 2023. Remdesivir was prescribed according to the national medical protocol in use at the time of admission.

## 2. Materials and Methods

We performed a retrospective study of COVID-19-positive children admitted to the Pediatric Infectious Diseases Department of the Clinical Hospital of Pneumology and Infectious Diseases in Brasov, Romania, between 1 January 2022, and 30 September 2023. Patients aged between 1 day and 17 years received remdesivir during hospitalization. Informed consent for treatment, data sharing, and publication was obtained at admission in all cases from the legal guardians. The patients were deidentified, and the study data were extracted from existing hospital administrative databases or electronic medical records by the primary author. All patients were tested for SARS-CoV-2 infection at admission, using either RT-PCR or rapid antigen testing. The data collected were age, sex, time from onset of symptoms to admission, comorbidities or previous conditions, severity of SARS-CoV-2 infection, clinical manifestations, admission to the pediatric ward or pediatric intensive care unit (PICU), need for oxygen administration, length of stay, and clinical course and outcome. We also included significant laboratory data: WBC, CRP, and liver and kidney function tests (alanine aminotransferase ALT and serum creatinine, respectively), measured at the beginning and end of remdesivir treatment. As the normal values of the laboratory analyses show some differences depending on the age of the patients and the laboratory where it was performed (in our case, the laboratory of the Pediatric Clinical Hospital in Brasov and the laboratory of the Clinical Hospital of Pneumology and Infectious Diseases in Brasov), to obtain a homogeneous evaluation of the changes in the analyses, a qualitative assessment was made of, respectively, a normal, low, or increased value. The severity or clinical form of COVID-19 was established according to the standard protocols used at the time of the patient’s admission [20,21]:

- mild form: general or upper respiratory tract symptoms, without manifestations evocative of pneumonia, without lung damage;
- medium form: patients with imaging-confirmed pneumonia, but without hypoxemia (with no respiratory impairment before the current illness);
- severe form: respiratory distress with oxygen saturation below 94% in atmospheric air and imaging abnormalities suggesting lung damage;
- critical form: patients with severe respiratory failure requiring ventilatory support, septic shock, or multiple organ dysfunction.

According to the same protocols, children with a history of chronic diseases (neurological pathology, genetic syndromes including trisomy 21, obesity, chronic cardiopulmonary diseases), immunocompromised children, and older adolescents (aged over 16 years) could be considered at high risk of severe disease.

Remdesivir doses were administered according to EMA recommendations: 5 mg/kg on the first day and 2.5 mg/kg on the following days [22].

## 3. Results

We identified 246 COVID-19-positive children who were admitted to our department between 1 January 2022 and 30 September 2023. Of these, 148 (60.16%) were younger than 1 year, and 98 (39.84%) were older than 1 year. The diagnosis of SARS-CoV-2 infection was obtained at admission using either the RT-PCR test or the rapid antigen test. Only 14 (5.69%) patients, representing our study group, received remdesivir (Table 1). In the remdesivir group, 7/14 (50%) cases presented various comorbidities (recurrent wheezing, congenital ichthyosis, congenital heart malformation) or had a history of acute, predominantly infectious conditions (acute tonsillitis, bronchiolitis, pneumonia, infectious mononucleosis). The interval from the onset of disease to hospitalization was less than 3 days, with five patients presenting the same day and six patients the day after the onset of symptoms (Table 2). No patient required initial admission to the PICU. The clinical picture was dominated by fever (13/14, 92.85%) and cough (8/14, 57.14%). Dyspnea was rare (4/14, 28.57%) and always associated with pneumonia, bronchiolitis, or laryngitis. Only 4/14 (28.57%) children had digestive symptoms (vomiting or diarrhea). The initial clinical picture was represented by convulsions in one patient and syncope in another (Table 3). During the clinical course, 13 children had documented pneumonia, one patient developed bronchiolitis, and another four had acute laryngitis, all associated with pneumonia. In two patients, bacterial coinfections were diagnosed by RT-PCR from the respiratory secretions: two cases of infection with *Streptococcus pneumoniae*, associated with *Haemophilus influenzae* in one case.

Regarding the laboratory data (Table 4), the WBC level was normal in 12/14 (85.71%) cases, with associated lymphopenia in 8/14 (57.14%). CRP levels were measured at admission in 11/14 (78.57%) patients and were elevated in only 2/11 (18.18%). Alanine aminotransferase (ALT) values at admission were measured in 12/14 (85.71%) patients and were normal in 11/12 (91.66%); only one child had elevated values, but he had also experienced a recent episode of infectious mononucleosis with associated hepatitis. Serum creatinine was normal on admission in all patients for whom it was measured (11/14, 78.57%).

Remdesivir treatment was initiated at the onset of hospitalization in 3/14 (21.43%) patients and after 1–6 days in the remaining 11/14 (78.57%). The duration of remdesivir treatment varied, from 1 day in a patient who required transfer to PICU the day after admission to 7 days in a patient who presented with a severe clinical form of COVID-19, with associated pneumonia, bronchiolitis, and coinfection with *S. pneumoniae*. Remdesivir was administered to most of the patients (8/14, 57.14%) for five days. The patients received other simultaneous therapies. Parenteral corticosteroid therapy was administered to 12/14 (85.71%) patients, and antibiotics were also given to 12/14. Remdesivir, cortisone, and antibiotic-associated therapy were applied to 10/14 (71.42%) patients.

The clinical course of patients treated with remdesivir was favorable in 12/14 (85.71%) patients, with discharge in an improved condition after variable periods of hospitalization (Table 5). Oxygen administration was required in just 2/14 (14.28%) cases: one with associated pneumonia and bronchiolitis and the second with associated pneumonia and laryngitis. The severity of COVID-19, as well as the need for associated antibiotic or cortisone treatment, influenced the duration of hospitalization. Two patients required transfer to the PICU: one for worsening bronchiolitis and the requirement of oxygen after 12 days of hospitalization and the other after displaying a rapid worsening of associated acute laryngitis after the first day of hospitalization.

## 4. Discussion

The mechanism of action of remdesivir functions by inhibiting viral replication through the premature termination of RNA transcription following binding to a viral RNA-dependent polymerase [12,23]. The antiviral effect of remdesivir on some RNAs was demonstrated by studies conducted before the SARS-CoV-2 pandemic. Furthermore, clinical studies have highlighted the usefulness and safety of using remdesivir in various contexts, including in Ebola virus infection [24,25].

For the treatment of SARS-CoV-2 infection in children, remdesivir is recommended for patients meeting certain conditions: they must be at least 12 years old and weigh at least 40 kg, the onset of symptoms must be at least 7 days ago, they must not need additional oxygen administration, and they must present risk factors for severe disease evolution [26]. The dosage differs according to the weight of the patient. For those weighing over 40 kg, 200 mg is administered on the first day and then 100 mg/day in a single dose. For patients weighing less than 40 kg (3–40 kg), the dose is calculated according to weight: 5 mg/kg on the first day and then 2.5 mg/day in a single dose (10,25). In adults, the initiation of remdesivir treatment in the first 7 days of the disease has been associated with a decrease in mortality at 28 days and a reduction in the need for mechanical ventilation [27].

Studies on remdesivir treatment in children and adolescents were carried out at the beginning of the COVID-19 pandemic in the context of its compassionate use [28,29]. Goldman et al. conducted a study on a group of 77 pediatric patients from six countries who underwent treatment with remdesivir for severe forms of COVID-19 between 21 March 2020, and 22 April 2020. The patients were of various ages, with a median of 14 years, and 79% had at least one comorbidity. Of these children, 90% required additional oxygen from the beginning, and 51% needed invasive ventilation. By day 28, 83% had recovered, and 73% were discharged. Of the children who required invasive ventilation, 80% recovered, and 67% were discharged. Of the four recorded deaths, three were interpreted as being caused by SARS-CoV-2 infection. The study highlighted the good level of tolerance to remdesivir therapy, the high recovery rate of the treated children, and a low incidence of serious adverse events (16%) [28].

Mendez-Echevarria et al. published a study conducted on eight infants and older children with severe COVID-19 who received remdesivir [29]. Six required admission to the PICU, five required mechanical ventilation (14 to 23 days), and one case required noninvasive ventilation. The evolution was favorable in seven patients. Patient 8 presented with multifactorial renal failure and died of COVID-19 and severe complications 10 days after remdesivir administration, initiated late after the onset of SARS-CoV-2 infection symptoms. No increase in liver enzymes was found in any patient.

A narrative analysis type of study, published in 2021 by La Tessa et al., discussed that at that time there were few studies on the use of remdesivir in the treatment SARS-CoV-2 infection in children, especially in severe or critical forms of the disease. They also noted the effectiveness of remdesivir therapy, administered as early as possible in the course of the disease, as well as the low rate of serious adverse events [30].

A study of a large number of children hospitalized with COVID-19 in the intensive care unit in 2020 found that most received therapies targeting the SARS-CoV-2 virus, including remdesivir, despite little data at the time on the use of new antiviral agents in pediatric patients [31]. Another multi-center study, conducted on 52 patients with COVID-19, highlighted that remdesivir treatment was generally well-tolerated; 82% of patients were discharged, and many showed clinical improvements based on a 7-point ordinal scale [32]. Adverse events were recorded in 21 patients, comprising acute renal failure, increased serum alanine aminotransferase values, hyperglycemia, and increased blood pressure.

Data from the specialized literature suggests that remdesivir treatment is safe, as demonstrated by the lack of adverse effects directly attributable to the drug, in the treatment of pediatric SARS-CoV-2 infections. Patients who received multiple doses of remdesivir had fewer symptoms and lower median World Health Organization Ordinal Scale scores for clinical improvement [12]. The use of remdesivir was also correlated with a reduced need for invasive mechanisms of additional oxygen administration. Less than a quarter of patients required an increase in oxygen support while receiving remdesivir [12,33,34].

In our geographical area, one paper analyzed the use of remdesivir in children. The study was performed in Bucharest and showed that the use of remdesivir in the treatment of COVID-19 in children was not associated with serious adverse reactions [34].

Our study evaluated the efficiency and safety of remdesivir treatment in 14 pediatric patients aged between 2 months and 15 years, who were diagnosed with mild (1 patient), moderate (11 patients), and severe (2 patients) forms of COVID-19. Remdesivir was used in only 5.69% of all children hospitalized with COVID-19, in similar proportions in infants and children over 1 year old (5.41% of hospitalized infants and 6.12% of hospitalized patients aged over 1 year). A high proportion of young infants received remdesivir (7/14, 50%), the youngest aged 2 months.

Hospitalization occurred early after the onset of symptoms in most patients. Only one child was hospitalized three days after onset. The speed with which the diagnosis of SARS-CoV-2 infection was established is notable, occurring in all cases on the day of admission, regardless of the test used for the diagnosis. This allowed the rapid establishment of treatment, including remdesivir, in most cases. In just two patients, the remdesivir treatment was initiated five and six days after the onset of symptoms due to the aggravation of symptoms attributed to SARS-CoV-2 infection. The presence of some comorbidities, along with young age and clinical form, determined the decision to treat with remdesivir. All patients were initially hospitalized in the pediatric ward, not the PICU.

Notably, the dominant clinical manifestations were uncharacteristic of COVID-19, namely, fever and cough, which are symptoms also found in other respiratory diseases of various etiologies. Testing for SARS-CoV-2 was imposed in a pandemic epidemiological context, a situation that must be considered in the future, to properly manage cases. The patients were also tested by RT-PCR of respiratory secretions for other respiratory, viral, and bacterial infections. No viral coinfections were identified, but bacterial coinfections were identified in two children: one with mono-infection with *S. pneumoniae* and one with double infection with *S. pneumoniae* and *H. influenzae*.

Only two patients, with severe clinical forms, required administration of oxygen with a simple face mask at the time of hospitalization. One, a 5-month-old infant with associated pneumonia and bronchiolitis, coinfection with *S. pneumoniae*, and a background of recurrent wheezing, was transferred to the PICU on the day of admission. The second patient who required oxygen from admission, with a medium clinical form of COVID-19 associated with bronchiolitis, evolved favorably, with discharge. This suggests the need to correctly evaluate the involvement of various associated pathologies in a certain evolutionary modality in patients with COVID-19.

The analysis of changes in serum leukocytes revealed interesting information. Most patients had a normal number of leukocytes, and lymphopenia, a marker of the severity of the SARS-CoV-2 infection, was identified in only some of the patients: one with a mild form, one with a severe form, and six with moderate forms of the disease. In only two cases was lymphopenia associated with leukopenia, in two patients with moderate forms of the disease. These findings strengthen the evidence from the specialized literature, according to which dynamic monitoring of the number of lymphocytes is required to assess the risk of severe evolution [35].

To identify possible adverse effects of remdesivir, the values of ALT and serum creatinine were analyzed at the initiation and completion of treatment. These two analyses were not performed at admission or at the end of treatment for all patients, for administrative reasons. The patients had normal ALT values at the beginning of antiviral treatment in most of the investigated cases; only one had elevated values. After completing the treatment with remdesivir, normal values were found in 9/12 patients, with increased values below two times the normal value in two patients. In the patient who had an increased value initially, it remained increased but decreased compared to the initial level. These data must be interpreted with caution because the patients simultaneously received other potentially liver-toxic medications, namely, paracetamol (acetaminophen), which is frequently used in the treatment of fever. Serum creatinine had normal values at admission in the patients for whom this analysis was performed and remained within normal limits even after the completion of the antiviral treatment, reflecting the lack of renal toxicity among the patients in our study group.

Patients in the study received etiological treatment for SARS-CoV-2 infection with remdesivir. The initiation of therapy with remdesivir fell within the interval recommended by specialist forums: in the first seven days from the onset of the disease. Twelve patients received remdesivir within three days of the onset of symptoms. This was due to their rapid presentation to the hospital, early diagnosis, and rapid assessment of the need for antiviral treatment. The duration of remdesivir treatment varied: eight patients received five days of treatment; five patients received between one and four days; only one patient, with severe COVID-19 and associated pneumonia and bronchiolitis, received remdesivir for seven days. No patient experienced a hypersensitivity reaction to remdesivir. All these findings suggest that both the timing of the therapeutic intervention and its duration represent important factors for the evolution of the disease.

The patients in the study group mostly benefited from other simultaneous treatments, comprising corticotherapy (12/14) and antibiotics (12/14). The associated therapies may have contributed to the favorable evolution observed in most patients, namely, the discharge of 12 of them, after variable periods of hospitalization due to COVID-19 and associated diseases.

Limitations: Our study had several limitations. In terms of the study type, our study was a retrospective, single-center study in which a small number of children patients received treatment with Remdesivir. In addition, the analysis was performed on the existing medical record, which was in some cases incomplete in terms of laboratory investigations. Due to the complex treatment administered to children with COVID-19, which also included corticotherapy and antibiotic therapy, the conclusions regarding the efficacy and amplitude of side effects of remdesivir as a single therapy should be used with precautions. Another limitation that should be mentioned in terms of treatment with Remdesivir in children is that each patient should be treated in concordance with the particularity of the case. The approach of combined therapy should be analyzed taking into consideration any aspects that could further influence the condition of the patient.

## 5. Conclusions

Our study contributes to the development of medical experience regarding the usefulness of remdesivir treatment in pediatric patients with COVID-19. Remdesivir contributed to the favorable evolution of SARS-CoV-2 infection in the majority of patients and was safe in terms of adverse effects. Further studies are required to support the efficiency, tolerability, and safety of remdesivir in children.

## Figures and Tables

**Table 1:** Distribution by gender and age group of pediatric patients diagnosed and admitted with COVID-19.

	Gender		Treatment with Remdesivir *n* (%)
	Male *n* (%)	Female *n* (%)	
Infants and newborns	66 (44.59%)	82 (55.41%)	8 (5.41%)
Children aged 1–17.9 years old	70 (71.43%)	28 (28.57%)	6 (6.12%)
Total	136 (55.28%)	110 (44.72%)	14 (5.69%)

**Table 2:** Epidemiological and clinical characteristics of pediatric patients diagnosed and admitted with COVID-19.

Patient	1	2	3	4	5	6	7	8	9	10	11	12	13	14
Age (Year, Month)	5 M	4 M	2 M	1 Y 1 M	1 Y 2 M	3 M	2 Y	15 Y	1 Y	4 Y	5 M	1 Y 10 M	2 M	5 M
Gender	M	M	M	F	F	F	M	M	M	M	M	F	M	F
Onset: Admission Interval (days)	1	Same day	Same day	1	Same day	1	2	Same day	2	Same day	1	3	1	1
PPH	Recurrent wheezing	-	*Enterococcus* spp. infection		Congenital ichthyosis	-	Acute angina Urinary infection	-	Bronchiolitis Pneumonia	-	-	Mononucleosis with hepatitis	Congenital heart malformation	-
Fever	X	X	-	X	X	X	X	X	X	X	X	X	X	X
Cough	X	X	X	X	X	-	X	-	X	-	-	X	-	-
Dyspnea	X	-	-	X	-	-	-	-	-	-	-	X	-	X
Nasal obstruction	-	-	X	-	-	-	-	-	-	-	-	-	X	-
Vomiting	-	-	-	-	-	X	X	-	X	-	-	-	-	-
Diarrhea	-	-	-	-	X	-	-	-	X	-	-	-	-	-
O_2_ administration	X	-	-	-	-	-	-	-	-	-	-	-	-	X
Other	-	-	-	-	-	-	-	Syncope		Convulsion Headache				

Legend: M—month, Y—year, PPH—Personal Pathological History, M—male, F—female, X—present, “-”—absent.

**Table 3:** Clinical characteristics of pediatric patients diagnosed and admitted with COVID-19.

Patient	1	2	3	4	5	6	7	8	9	10	11	12	13	14
Clinical form of COVID-19														
Mild								X						
Medium		X	X	X	X	X	X		X	X	X	X	X	
Severe	X													X
Associated diseases														
Pneumonia	X	X	X	X	X	X	X		X	X	X	X	X	
Bronchiolitis	X													
Laryngitis		X		X								X		X
Others			Conjunctivitis Alergo-dermitis			Anemia	Anemia							
Admitted to ICU														
	NO	NO	NO	NO	NO	NO	NO	NO	NO	NO	NO	NO	NO	NO
SARS-CoV-2 Infection Confirmation														
RT-PCR	X		X											X
Rapid Antigen Test		X		X	X	X	X	X	X	X	X	X	X	

Legend: ICU—Intensive Care Unit. X—present, “NO”—Not admitted to ICU.

**Table 4:** Laboratory data and therapeutic data of pediatric patients diagnosed and admitted with COVID-19.

Patient	1	2	3	4	5	6	7	8	9	10	11	12	13	14
Leucocytes	N	N	N	N	N	D	N	N	N	N	N	N	D	N
Lymphocytes	N	N	N	N	D	D	D	D	N	D	D	N	D	D
CRP	N	D	N	N/A	N/A	N	N	N	I	N	N	N/A	N	N
ALT (TGP) at admission	N	I	N	N/A	N/A	N	N	N	N	N	N	I	N	N
Serum creatinine	N	N	N	N/A	N/A	N	N	N	N/A	N	N	N	N	N
Onset: Administration of Remdesivir interval (Days)	6	1	Same day	1	1	2	3	Same day	3	Same day	2	5	1	2
No of days of Remdesivir administration	7	5	2	1	5	5	5	3	4	5	5	4	5	5
Corticotherapy	Yes	Yes	Yes	Yes	No	Yes	Yes	Yes	Yes	Yes	Yes	Yes	No	Yes
Antibiotics	Yes	Yes	Yes	Yes	Yes	No	Yes	No	Yes	Yes	Yes	Yes	Yes	Yes

Legend: CRP—C Reactive Protein, N—Normal Value, I—increased value, D—decreased value, N/A—not assessed.

**Table 5:** Evolutive data of pediatric patients diagnosed and admitted with COVID-19.

Patient	1	2	3	4	5	6	7	8	9	10	11	12	13	14
Clinical evolution after Remdesivir administration														
Improvement and discharge from hospital		X	X		X	X	X	X	X	X	X	X	X	X
COVID-19 worsening and transfer	-	-	-	-	-	-	-	-	-	-	-	-	-	-
Worsening of the associated disease and transfer to PICU	X Bronchiolitis		X Laryngitis											
Hospitalization days														
n	12	5	2	1	8	7	8	3	14	5	5	6	9	6
ALT (TGP)—at discharge														
n	N	N	N	N/A	I	I	N	N	N	N/A	N	D	N	N
Serum creatine														
n	N	N	N	N/A	N	N	N	N	N	N/A	N	N	N	N

Legend: N—Normal Value, I—increased value, D—decreased value, N/A—not assessed, X—present.

## Data Availability

Data are contained within the article.

## References

[1] Choi S., Choi J.H., Yun K.W. (2022). Therapeutics for the Treatment of Coronavirus Disease 2019 in Children and Adolescents. Clin. Exp. Pediatr. Online.

[2] Fraile Navarro D., Tendal B., Tingay D., Vasilunas N., Anderson L., Best J., Burns P., Cheyne S., Craig S.S., Erickson S.J. (2021). Clinical Care of Children and Adolescents with COVID-19: Recommendations from the National COVID-19 Clinical Evidence Taskforce. Med. J. Aust..

[3] Lee H., Choi S., Park J.Y., Jo D.S., Choi U.Y., Lee H., Jung Y.T., Chung I.H., Choe Y.J., Kim J.Y. (2022). Analysis of Critical COVID-19 Cases among Children in Korea. J. Korean Med. Sci..

[4] Woodruff R.C., Campbell A.P., Taylor C.A., Chai S.J., Kawasaki B., Meek J., Anderson E.J., Weigel A., Monroe M.L., Reeg L. (2021). Risk Factors for Severe COVID-19 in Children. Pediatrics.

[5] Kompaniyets L., Agathis N.T., Nelson J.M., Preston L.E., Ko J.Y., Belay B., Pennington A.F., Danielson M.L., DeSisto C.L., Chevinsky J.R. (2021). Underlying Medical Conditions Associated with Severe COVID-19 Illness among Children. JAMA Netw. Open.

[6] Khalil A., Mohamed A., Hassan M., Magboul S., Ali H., Elmasoudi A., Ellithy K., Qusad M., Alhothi A., Al Maslamani E. (2023). Efficacy and Safety of Remdesivir in Hospitalized Pediatric COVID-19: A Retrospective Case-Controlled Study. Ther. Clin. Risk Manag..

[7] Dulek D.E., Fuhlbrigge R.C., Tribble A.C., Connelly J.A., Loi M.M., El Chebib H., Chandrakasan S., Otto W.R., Diorio C., Keim G. (2020). Multidisciplinary Guidance Regarding the Use of Immunomodulatory Therapies for Acute COVID-19 in Pediatric Patients. J. Pediatr. Infect. Dis. Soc..

[8] National Institute for Health and Care Excellence (2021). COVID-19 Rapid Guideline: Managing COVID-19 [Internet].

[9] Information on COVID-19 Treatment, Prevention and Research. https://www.covid19treatmentguidelines.nih.gov/.

[10] World Health Organization (2022). Therapeutics and COVID-19: Living Guideline [Internet].

[11] Hall M.D., Anderson J.M., Anderson A., Baker D., Bradner J., Brimacombe K.R., Campbell E.A., Corbett K.S., Carter K., Cherry S. (2021). Report of the National Institutes of Health SARS-CoV-2 Antiviral Therapeutics Summit. J. Infect. Dis..

[12] Samuel A.M., Hacker L.L., Zebracki J., Bogenschutz M.C., Schulz L., Strayer J., Vanderloo J.P., Cengiz P., Henderson S. (2023). Remdesivir Use in Pediatric Patients for SARS-CoV-2 Treatment: Single Academic Center Study. Pediatr. Infect. Dis. J..

[13] Chiotos K., Hayes M., Kimberlin D.W., Jones S.B., James S.H., Pinninti S.G., Yarbrough A., Abzug M.J., MacBrayne C.E., Soma V.L. (2020). Multicenter Interim Guidance on Use of Antivirals for Children with COVID-19/SARS-CoV-2. J. Pediatr. Infect. Dis. Soc..

[14] FDA Approves First Treatment for COVID-19. https://www.fda.gov/news-events/press-announcements/fda-approves-first-treatment-covid-19.

[15] Elena Kostadinova DIMITROVA Veklury—European Medicines Agency. https://www.ema.europa.eu/en/medicines/human/EPAR/veklury.

[16] Korea Disease Control and Prevention Agency (2022). Guidance on the Use of COVID-19 Treatment [Internet].

[17] Beigel J.H., Tomashek K.M., Dodd L.E., Mehta A.K., Zingman B.S., Kalil A.C., Hohmann E., Chu H.Y., Luetkemeyer A., Kline S. (2020). Remdesivir for the Treatment of COVID-19—Preliminary Report. N. Engl. J. Med..

[18] Touafchia A., Bagheri H., Carrié D., Durrieu G., Sommet A., Chouchana L., Montastruc F. (2021). Serious Bradycardia and Remdesivir for Coronavirus 2019 (COVID-19): A New Safety Concerns. Clin. Microbiol. Infect..

[19] Kautsch K., Wiśniowska J., Friedman-Gruszczyńska J., Buda P. (2023). Evaluation of the Safety Profile and Therapeutic Efficacy of Remdesivir in Children with SARS-CoV-2 Infection—A Single-Center, Retrospective, Cohort Study. Eur. J. Pediatr..

[20] Treatment Protocol for Infection with SARS-CoV-2 Virus—January 2022 (Order of the Ministry of Health—Romania). https://legislatie.just.ro/Public/DetaliiDocument/250463.

[21] Treatment Protocol for Infection with SARS-CoV-2 Virus—June 2022 (Order of the Ministry of Health—Romania). https://lege5.ro/gratuit/geztonzzgq3ts/protocolul-de-tratament-al-infectiei-cu-virusul-sars-cov-2-din-22062023.

[22] Veklury (Remdesivir) an Overview of Veklury and Why It Is Authorised in the EU. https://www.ema.europa.eu/en/documents/overview/veklury-epar-medicine-overview_en.pdf.

[23] Ko W.-C., Rolain J.-M., Lee N.-Y., Chen P.-L., Huang C.-T., Lee P.-I., Hsueh P.-R. (2020). Arguments in Favour of Remdesivir for Treating SARS-CoV-2 Infections. Int. J. Antimicrob. Agents.

[24] Yalçın N., Demirkan K. (2020). COVID-19 and Remdesivir in Pediatric Patients: The Invisible Part of the Iceberg. Pediatr. Res..

[25] Dörnemann J., Burzio C., Ronsse A., Sprecher A., De Clerck H., Van Herp M., Kolié M.-C., Yosifiva V., Caluwaerts S., McElroy A.K. (2017). First Newborn Baby to Receive Experimental Therapies Survives Ebola Virus Disease. J. Infect. Dis..

[26] Fact Sheet for Health Care Providers Emergency Use Authorization (EUA) of Remdesivir (GS-5734TM). https://www.fda.gov/media/137566/download.

[27] Hussain Alsayed H.A., Saheb Sharif-Askari F., Saheb Sharif-Askari N., Hussain A.A.S., Hamid Q., Halwani R. (2021). Early Administration of Remdesivir to COVID-19 Patients Associates with Higher Recovery Rate and Lower Need for ICU Admission: A Retrospective Cohort Study. PLoS ONE.

[28] Goldman D.L., Aldrich M.L., Hagmann S.H.F., Bamford A., Camacho-Gonzalez A., Lapadula G., Lee P., Bonfanti P., Carter C.C., Zhao Y. (2021). Compassionate Use of Remdesivir in Children with Severe COVID-19. Pediatrics.

[29] Méndez-Echevarría A., Pérez-Martínez A., Gonzalez del Valle L., Ara M.F., Melendo S., Ruiz de Valbuena M., Vazquez-Martinez J.L., Morales-Martínez A., Remesal A., Sándor-Bajusz K.A. (2020). Compassionate Use of Remdesivir in Children with COVID-19. Eur. J. Pediatr..

[30] Tessa A., Motisi M., Marseglia G., Cardinale F., Licari A., Manti S., Tosca M., Miraglia M., Giudice D., De Filippo M. (2021). Use of Remdesivir in Children with COVID-19 Infection: A Quick Narrative Review. Acta Biomed..

[31] Schuster J.E., Halasa N.B., Nakamura M., Levy E.R., Fitzgerald J.C., Young C.C., Newhams M.M., Bourgeois F., Staat M.A., Hobbs C.V. (2022). A Description of COVID-19-Directed Therapy in Children Admitted to US Intensive Care Units 2020. J. Pediatr. Infect. Dis. Soc..

[32] Gilead Sciences A Phase 2/3 Single-Arm, Open-Label Study to Evaluate the Safety, Tolerability, Pharmacokinetics, and Efficacy of Remdesivir (GS-5734TM) in Participants from Birth to <18 Years of Age with COVID-19. https://clinicaltrials.gov/ct2/show/NCT04431453.

[33] Gottlieb R.L., Vaca C.E., Paredes R., Mera J., Webb B.J., Perez G., Oguchi G., Ryan P., Nielsen B.U., Brown M. (2021). Early Remdesivir to Prevent Progression to Severe COVID-19 in Outpatients. N. Engl. J. Med..

[34] Jugulete G., Luminos M., Pavelescu C., Merișescu M.M. (2023). Remdesivir Efficacy and Tolerability in Children with COVID-19-Associated Allergic Comorbidities such as Asthma, Allergic Rhinitis, and Atopic Dermatitis. Children.

[35] Ghizlane E.A., Manal M., Abderrahim E.K., Abdelilah E., Mohammed M., Rajae A., Amine B.M., Houssam B., Naima A., Brahim H. (2021). Lymphopenia in COVID-19: A Single Center Retrospective Study of 589 Cases. Ann. Med. Surg..

